# Neuroprotective Effect of Artichoke-Based Nanoformulation in Sporadic Alzheimer’s Disease Mouse Model: Focus on Antioxidant, Anti-Inflammatory, and Amyloidogenic Pathways

**DOI:** 10.3390/ph15101202

**Published:** 2022-09-28

**Authors:** Heba A. S. El-Nashar, Haidy Abbas, Mariam Zewail, Mohamed H. Noureldin, Mai M. Ali, Marium M. Shamaa, Mohamed A. Khattab, Nehal Ibrahim

**Affiliations:** 1 Pharmacognosy Department, Faculty of Pharmacy, Ain Shams University, Cairo 11566, Egypt; 2 Department of Pharmaceutics, Faculty of Pharmacy, Damanhour University, Damanhour 22514, Egypt; 3 Department of Biochemistry, Division of Clinical and Biological Sciences, College of Pharmacy, Arab Academy for Science, Technology and Maritime Transport, Alexandria P.O. Box 1029, Egypt; 4 Department of Pharmaceutics, Division of Pharmaceutical Sciences, College of Pharmacy, Arab Academy for Science, Technology and Maritime Transport, Alexandria P.O. Box 1029, Egypt; 5 Department of Cytology and Histology, Faculty of Veterinary Medicine, Cairo University, Cairo 12211, Egypt

**Keywords:** artichoke bracts, UPLC-ESI-MS/MS, antioxidant, anti-Alzheimer, solid lipid nanoparticles, chitosan

## Abstract

The vast socio-economic impact of Alzheimer’s disease (AD) has prompted the search for new neuroprotective agents with good tolerability and safety profile. With its outstanding role as antioxidant and anti-inflammatory, alongside its anti-acetylcholinesterase activity, the artichoke can be implemented in a multi-targeted approach in AD therapy. Moreover, artichoke agricultural wastes can represent according to the current United Nations Sustainable Development goals an opportunity to produce medicinally valuable phenolic-rich extracts. In this context, the UPLC-ESI-MS/MS phytochemical characterization of artichoke bracts extract revealed the presence of mono- and di-caffeoylquinic acids and apigenin, luteolin, and kaempferol *O*-glycosides with remarkable total phenolics and flavonoids contents. A broad antioxidant spectrum was established in vitro. Artichoke-loaded, chitosan-coated, solid lipid nanoparticles (SLNs) were prepared and characterized for their size, zeta potential, morphology, entrapment efficiency, release, and ex vivo permeation and showed suitable colloidal characteristics, a controlled release profile, and promising ex vivo permeation, indicating possibly better physicochemical and biopharmaceutical parameters than free artichoke extract. The anti-Alzheimer potential of the extract and prepared SLNs was assessed in vivo in streptozotocin-induced sporadic Alzheimer mice. A great improvement in cognitive functions and spatial memory recovery, in addition to a marked reduction of the inflammatory biomarker TNF-α, β-amyloid, and tau protein levels, were observed. Significant neuroprotective efficacy in dentate Gyrus sub-regions was achieved in mice treated with free artichoke extract and to a significantly higher extent with artichoke-loaded SLNs. The results clarify the strong potential of artichoke bracts extract as a botanical anti-AD drug and will contribute to altering the future medicinal outlook of artichoke bracts previously regarded as agro-industrial waste.

## 1. Introduction

Alzheimer’s disease (AD) is responsible for about 70% of dementia cases, by which more than 130 million people will probably be afflicted by 2050 [1]. The huge socio-economic impact of AD has prompted the search for neuroprotective agents that can prevent or delay the initiation and progression of neurodegeneration with minimum adverse effects. In this context, the search for neuroprotective agents from phenolic-rich dietary sources is worth consideration, taking into account their good tolerability and safety profile [2]. The aggregation of β-amyloid peptide (Aβ) and tau protein hyperphosphorylation are critical neuropathological features of AD pathogenesis [3]. Moreover, neuroinflammation, oxidative stress, and a decrease in brain acetylcholine levels play central roles in cognitive impairment in AD [2].

*Cynara cardunculus* L. (globe artichoke) is a member of the small genus *Cynara* (Compositae) [4]. Artichoke is a fundamental ingredient in the Mediterranean diet and nowadays cultivated in many countries for its edible flower heads (capitula) because of their sensory and nutritional quality and health promoting properties [5]. During the last few years, Egypt has increased its production of artichoke to almost 310 kt in 2020 [6]. The production of artichoke is accompanied by the generation of substantial amounts of polyphenol-rich wastes (up to 85% of the aerial biomass) which, according to the current United Nations Sustainable Development goals, evoked much interest for their value as source of bioactive phytochemicals, food, and energy [5,7,8].

Artichoke was traditionally used for hepato-biliary diseases and digestive disorders [9]. Being rich in caffeoylquinic acids, flavonoids, anthocyanins, and sesquiterpene lactones [10], artichoke is endowed with a variety of pharmacological properties, including cytoprotective, antioxidant, anti-inflammatory, hepatoprotective, hypocholesterolemic, hypoglycemic, and anticancer activities [9,10,11]. With its recently established acetylcholinesterase inhibitory activity [12], alongside its outstanding dual role as antioxidant and anti-inflammatory [10,13], artichoke could be a promising starting material for developing multi-targeted neuroprotective treatments. Although acetylcholinesterase has been the most common pharmacological target in AD treatment, the interplay of other neurotransmitters cannot be underestimated viz. disturbed serotonergic, adrenergic and glutamatergic systems [14]. Therefore, the in vivo assessment could yield more reliable information to ascertain the anti-Alzheimer potential of plant extracts containing several bioactive constituents working synergistically and with multiple mechanisms of action that can better cover all aspects of AD pathogenesis.

Nevertheless, the medicinal use of natural products is challenged by many physicochemical and biopharmaceutical obstacles, including poor solubility and/or stability, poor bioavailability, high metabolism, short half-life, and extensive plasma protein binding [14]. These challenges may be suitably addressed by nanoformulation designs which can improve the overall therapeutic performance of natural products [15].

The aim of the present study was to assess the neuroprotective potential of artichoke bracts extract (ART) and the extract formulated in chitosan-coated solid lipid nanoparticles (SLNs) in vivo in streptozotocin-induced Alzheimer mice. The phytochemical composition of ART was profiled by UPLC-ESI-MS/MS and its antioxidant capabilities were evaluated via different in vitro models.

## 2. Results and Discussion

### 2.1. Assessment of Total Phenolic Content (TPC) and Total Flavonoids Content (TFC)

The TPC can mirror the antioxidant potential of plant extracts [16]. The TPC of ART, calculated as gallic acid equivalent (R^2^ = 0.9941), was 44.87 ± 1.88 µg GAE/mg dry extract, well above the TPC previously reported in artichoke bracts and whole inflorescence crude extracts and comparable to artichoke stems [10,17]. The TFC (R^2^ = 0.9988) was 2.33 ± 0.33 µg quercetin equivalent (QE)/mg dry extract, comparable to previous reports of artichoke crude extracts [10].

### 2.2. Metabolite Profiling via UPLC-ESI-MS/MS

UPLC-ESI-MS/MS analysis of ART was conducted to explore its phytochemical composition. Figure S1 (Supplementary Material) represents the total ion chromatogram. A total of 16 phenolic compounds, mainly flavonoid glycosides and phenolic acids, were tentatively identified by comparison of their MS characteristics and fragmentation pattern with previously reported data on *C. cardunculus* (Table 1). Flavonoid glycosides demonstrated the predominant class of metabolites identified in ART (Table 1). The hexose and rutinose conjugates of flavonoids exemplify the most common. Interestingly, apigenin detected in four of the identified metabolites (**7**–**9** and **14**), was reported to exert broad neuroprotective effects via its potent anti-inflammatory attributes, protective effects against caspase-3/7 mediated apoptosis and modulating spontaneous Ca^2+^ signals [18]. Additionally, kaempferol in (**11**) may improve cognitive function through its inhibitory action on acetylcholinesterase and modulatory effect of endogenous antioxidants [19]. Furthermore, the detected flavone luteolin in (**10** and **15**) may provide protection against AD by its influence on neuroinflammation, brain insulin resistance, and Aβ deposition [20]. Finally, the flavonol quercetin in (**13**) was reported [21] to interfere with the formation of Aβ plaques, destabilize formed fibril and inhibit β-secretase-1.

Four phenolic acids were identified in ART (**1, 3, 4** and **5**). Similar pseudo-molecular ion peaks [M−H]^−^ were observed for compounds (**4**) and (**5**) at *m*/*z* 515 suggesting the same molecular formula of C_25_H_24_O_12_. Both compounds were identified as di-caffeoylquinic acid isomers with typical fragment ions; *m*/*z* 353 equivalent to the loss of a caffeoyl moiety [M−H-caffeoyl]^−^, 191 due to loss of 2 caffeoyl moieties [M−H-2 caffeoyl]^−^ and 179 corresponding to caffeic acid. Chlorogenic acid is reported to have a neuroprotective power attributed to its anti-acetylcholinesterase and antioxidant properties [22]. The rich phenolic profile of ART was encouraging to assess its in vitro antioxidant properties.

### 2.3. Determination of ART Antioxidant Potential

The antioxidant potential was reported to rely mainly on phenolics content and can be interconnected with a wide range of pharmacological activities [25,30]. Generally, the antioxidants are classified into three mechanistic categories, including single electron transfer (SET), hydrogen atom transfer (HAT), and metal chelation [30,31]. Metal chelation assays can be considered an essential tool for screening the antioxidant and neuroprotective activity of phytoconstituents. Transition metal ions potentiate neurological damage via two pathways. First, the presence of transition metal ions e.g., iron provides the suitable pro-aggregating environment necessary for the formation of Amyloid-β plaques and induction of neurotoxicity [2]. Second, catalyzing transition metal ions, such as ferrous ions, could activate a Fenton reaction, inducing lipid peroxidation [32]. Therefore, there is a rationale to use metal ion chelation as a therapeutic approach in AD. ART showed a promising metal chelation capacity of ferrous ions (22.72 ± 2.71 μM EDTA eq/mg extract).

The ABTS assay characterizes the SET mechanism [33] and evaluates the antioxidant properties of lipophilic and hydrophilic phytoconstituents. ART showed interesting ABTS radical scavenging capacity of 490.76 ± 18.64 μM Trolox eq/mg extract, well above the reported ABTS scavenging capacity of different artichoke cultivars, hybrids [25] and wastes extracted with different solvents [34]. The ORAC assay represents the HAT mechanism. ART displayed an ORAC of 1125.49 ± 111.87 μM Trolox eq/mg extract, significantly higher than that reported by Turkiewicz et al. for artichoke [25].

The DPPH assay represents two mechanistic categories, SET and to lesser extent HAT [35]. ART showed a remarkable DPPH radical scavenging capacity (246.08 ± 30.15 μM Trolox eq/mg extract) superior to the previously reported data of artichoke wastes [34]. ART revealed a broad antioxidant spectrum, assessed with several methods, affording possible protection against neurological oxidative damage.

Due to the promising in vitro antioxidant potential of the prepared artichoke bracts extract, in vivo evaluation of its neuroprotective activity against AD will be implemented orally, taking into consideration its recently-reported acetylcholinesterase inhibitory activity [12]. Consequently, a delivery system is required to improve the poor physicochemical properties of the extract phytoconstituents, namely, (a) increase their aqueous solubility and absorption, in addition to (b) protecting the phenolic constituents from degradation in the digestive tract. In brief, this delivery system finally aims to transport sufficient amounts of the extract phytoconstituents to the brain as the site of action through the oral route. In this context, encapsulating the extract in a nano-lipid formulation could prove beneficial.

### 2.4. Preparation of Artichoke Loaded SLNs

The choice of SLNs was based on their several merits as they have high drug loading capacity, are biocompatible, physically stable, and can protect drugs against degradation [36,37]. Also, they can be loaded with both hydrophilic and lipophilic drugs [38]. SLNs have numerous applications in delivery of phytoconstituents to manage different types of chronic diseases, such as cancer, neurodegeneration, and diabetes [38]. SLNs were prepared by the solvent evaporation method using GMS and either Poloxamer 407 or Tween 80 as surfactants. In order to increase the SLNs mucoadhesive properties, improve the nanocarrier permeation through the gastrointestinal mucosa, and decrease their burst release, SLNs were coated with CS [38,39].

### 2.5. Particle Size, Zeta Potential and Entrapment Efficiency

Colloidal characteristics of different artichoke-loaded SLN formulations are listed in Table 2. The effect of surfactant type was investigated; where SLN containing Poloxamer 407 showed a significantly (*p* < 0.05) smaller particle size compared to those containing Tween 80 with and without CS coating. These findings are in accordance with the results previously reported by Anchan et al. [39] and may be attributed to the increased deposition of CS on SLNs’ surface triggered by the decreased solubility in presence of Tween 80 compared with Poloxamer 407 [39]. CS-coated SLNs (F3, F4) showed a significant increase in particle size compared to uncoated SLNs (F1, F2). This is in agreement with previously reported results [37,40].

The zeta potential of Poloxamer 407 containing SLNs (F1) and Tween 80 containing SLNs (F2) were −26.3 and −32.2 meV, respectively. Electrostatic interaction between the positively charged chitosan chains and the negatively charged lipids in SLNs affords effective coating of SLNs with CS [41]. Zeta potential was inverted from the negative side in uncoated SLNs (−26.3, −32.5 meV) to the positive side in CS-SLNs (19.25, 22.4 meV) (Table 2). Inversion of the negative surface charge of SLNs to positive charge upon coating with CS was formerly described [37,40,42,43,44,45].

Poloxamer 407 containing formulations, such as F1 and F3, had higher % EE compared to F2 and F4 containing Tween 80. This might be attributed to the greater solubilizing effects of Poloxamer 407 compared to Tween 80 [39]. Furthermore, CS-SLNs such as F3 had higher EE % compared to uncoated SLNs (F1), namely 79% and 74 %, respectively. This can be ascribed to the influence of CS coating on increasing drug loading % and EE % [40,46,47].

### 2.6. Morphology of Uncoated and Coated SLNs

**Figure 1:**
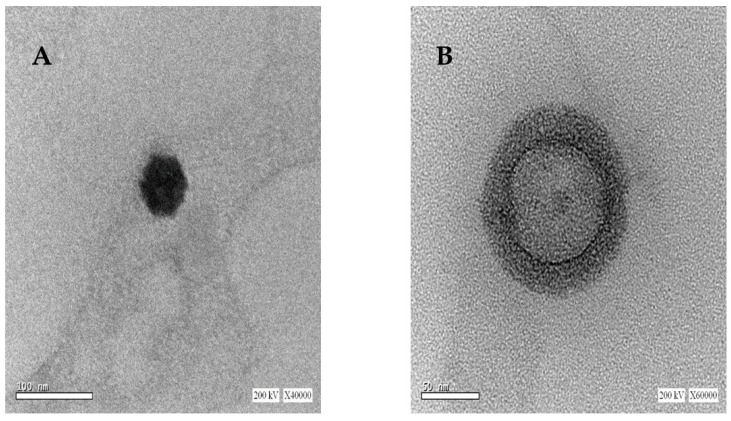
TEM micrographs of Poloxamer 407 based (**A**) uncoated SLNs and (**B**) CS-coated SLNs.

Due to the superior colloidal properties of the SLNs obtained by Poloxamer 407, their morphological examination (uncoated and CS-coated SLNs) was undertaken by TEM, which revealed that the prepared SLNs had a spherical uniform shape, as shown in Figure 1A. A successful CS coating layer can be visually confirmed in Figure 1B with a relative increase in particle size compared to uncoated SLNs.

### 2.7. In Vitro Release Study

**Figure 2:**
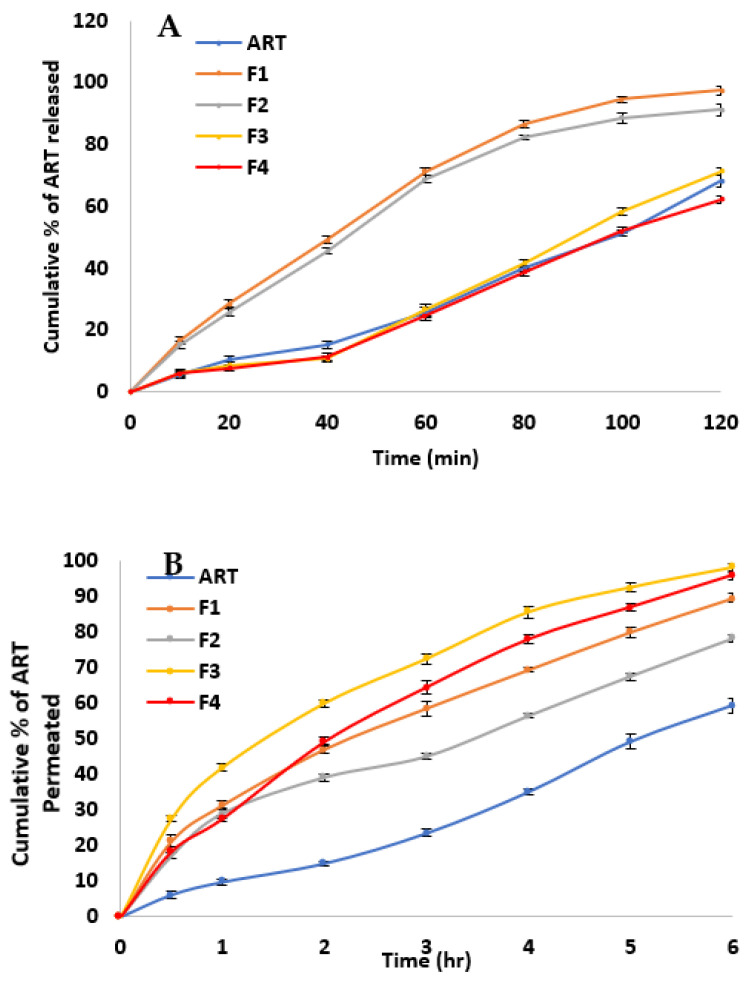
(**A**) Cumulative percentage of ART released in acidic medium and (**B**) Cumulative percentage of ART permeated through the intestinal mucosa for the prepared SLNs (F1: Poloxamer 407/uncoated—F2: Tween 80/uncoated—F3: Poloxamer 407/chitosan coated—F4: Tween 80/chitosan coated).

Figure 2A shows the release profiles of ART from different formulations in 0.1 N HCl to simulate the acidic conditions in the stomach. ART showed a poor release profile (68% in 120 min). This may be attributed to the poor aqueous solubility of the extract phytoconstituents. Encapsulation of ART into SLNs resulted in controlled but significantly (*p* < 0.05) faster release profiles than ART alone, with SLNs formulated with Poloxamer 407 showing faster release in comparison to SLNs formulated with Tween 80 (97.5 ± 0.92 and 91.2 ± 0.87 for F1 and F2, respectively). This may be attributed to their smaller size, as seen from size measurements, higher surface area, and the higher solubilizing power of Poloxamer 407 compared to Tween 80.

Coating of ART loaded SLNs with CS resulted in slowing the ART release and decreasing the burst effect. This result is in agreement with that of Zewail et al. [37], who reported the ability of CS-SLNs to extend the release period from 72 h in uncoated SLNs to 168 h in CS-SLNs, in addition to their ability to significantly decrease the burst effect. Again, SLNs formulated with Poloxamer 407 show comparatively faster release in comparison to SLNs formulated with Tween 80 (71.2 ± 1.12 and 62.1 ± 1.02 for F3 and F4, respectively).

The release kinetics of different SLNs formulations were fitted to zero-order, first-order, and Higuchi models and their correlation coefficients (R2) are listed in Table 2. Results show that in comparison to the extract whose release best fitted the zero-order model (R2 = 0.979), all the SLNs correlated well with the Higuchi model, indicating a diffusion-based release mechanism of phytoconstituents from the lipid matrix of the SLNs.

### 2.8. Ex Vivo Permeation Studies using Goat Intestinal Mucosa

The permeation profiles of different ART formulations through goat intestinal mucosa are illustrated in Figure 2B. ART showed a poor permeation profile (<60% in 6 h). This may be attributed to the low aqueous solubility or metabolism [48]. When encapsulated in SLNs, ART phytoconstituents showed higher mucosal permeation results. This may be due to the protective effect of SLNs against phytoconstituents degradation by different gut metabolizing proteins and/or their solubilizing enhancing effect as observed in the release study.

However, CS coating showed superior permeation results compared to uncoated SLNs and ART, although the release test showed a slower release profile. This may be attributed to the reported mucoadhesive properties of CS, increasing the attraction of the coated SLNs to the mucosal surface, which may allow the formation of local higher concentration gradients at the mucosal barrier surface, and consequently faster permeation. This faster permeation may prove beneficial in protecting the phytoconstituents from the harsh metabolizing environment of the oral route. These findings are in line with those previously reported by Anchan et al. [39], who reported the ability of CS-coated insulin-loaded SLNs to enhance insulin intestinal permeation compared to uncoated SLNs and free insulin [39].

Based on the aforementioned results, the ART extract and formulation F3 (CS-coated ART-loaded SLNs) were selected for the evaluation of their in vivo performance as promising antioxidant and neuroprotective agents. Selection of an AD animal model is suitable as AD pathophysiology entails inflammation with prominent ROS levels elevation and neurological damage caused by β-amyloid peptide (Aβ) aggregation and tau protein hyperphosphorylation.

### 2.9. In Vivo Studies

**Figure 3:**
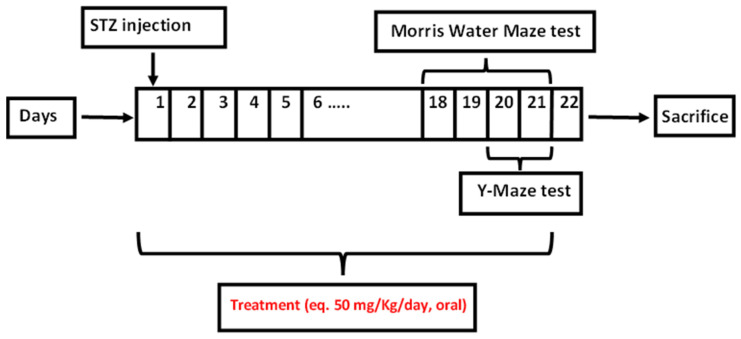
Schematic representation of experimental design illustrating drug dose, duration of the experiment and on which days behavioral tests were carried out.

As depicted in Figure 3, induction of AD was achieved by STZ (ICV injection) due to its reported cytotoxicity against insulin-producing beta cells in CNS and periphery. STZ can induce structural, neurochemical, as well as behavioral changes in animals similar to those encountered in sporadic Alzheimer’s disease (SAD). SAD is the most common form of dementia in the elderly, which is accompanied by progressive neurodegeneration of the CNS [49,50,51]. STZ is mainly taken up by glucose receptors, resulting in insulin desensitization, downregulation of insulin receptor substrate, and the eventual reduction of glucose metabolism which results in neuron degeneration [50].

#### 2.9.1. Y-Maze Test

**Figure 4:**
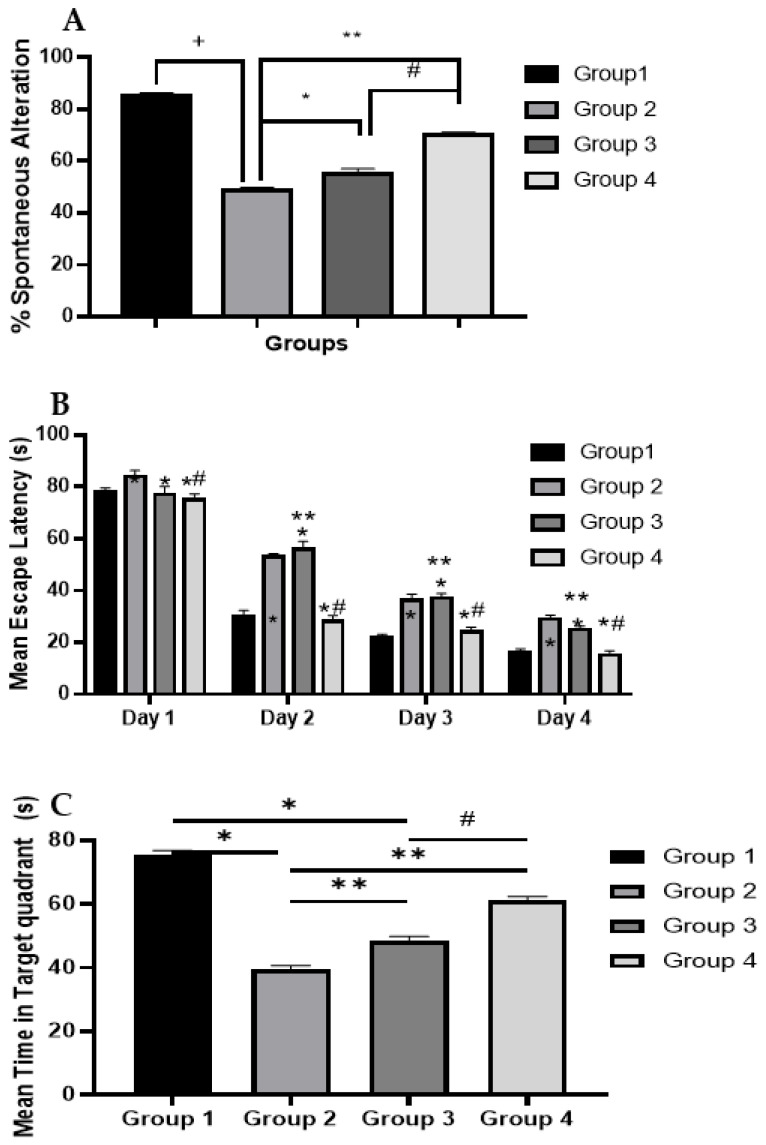
Behavioral assessment of the effect of ART on (**A**) the percentage of spontaneous alternation, (**B**) mean escape latency in MWM and (**C**) time spent in target quadrant in MWM. Statistical analyses are performed using one-way analysis of variance (ANOVA) followed by Tukey post-hoc test. +: Statistically significant different from the normal control group (Saline) at *p* < 0.05, * and **: Statistically significant different from the positive control group (STZ, 3 mg/kg) at *p* < 0.05, #: Statistically significant different from the ART group (50 mg/kg) at *p* < 0.05. ART-Nanoparticles (equivalent to 50 mg/kg).

Short-term memory in mice was assessed by evaluating the percentage of spontaneous alternation [50], as demonstrated in Figure 4A. The decrease in spontaneous alternation percentage of the positive control (group 2) compared to negative control (group 1) is consistent with previous reports that indicate short-term memory impairment [50,51,52] due to STZ ICV injection. The alternation percentage noted in the positive control (group 2) was statistically lower than that found in other treatment groups (groups 3 and 4), which is an indication of the neuroprotective potential of ART. Group 4 treated with CS-SLNs showed a statistically significant higher alternation percentage than the group treated with free ART (group 3), which again highlights the potential role of nanoformulation in the protection and successful delivery of the phytoconstituents. This result is also supported by the ex vivo permeation results where the nanoformulation showed higher permeation than the free ART extract.

#### 2.9.2. Morris Water Maze

Mean escape latency (MEL) values for different treatment groups are shown in Figure 4B. MEL results of the positive control (group 2) were in line with previous results that show the ability of STZ ICV injection to lead to memory and learning impairments, which were demonstrated by statistically significant suboptimal performance in comparison to negative control (group 1) on the test days [50,51]. Mice treated with ART (group 3) displayed a gradual decrease in mean escape latency (MEL) values. However, the results were not statistically significant from positive control except at day 4. This result indicates that ART may have a beneficial effect on the mice visuospatial memory and learning capacity but the amount reaching the brain is not sufficient to retrieve the neurological damage. On the other hand, the ART-loaded CS-coated SLNs formulation was able to improve the visuospatial memory and learning capacity of the mice and was the only treatment modality that showed a statistically insignificant change from the negative control (group 1). This improvement in ART performance is in agreement with the Y-maze results and may indicate the ability of the delivery system to create higher brain concentrations of the phytoconstituents, as demonstrated by better performance. This may be accredited to the effect of CS-coated SLNs in improving the stability and intestinal absorption properties of ART and eventually its pharmacological effects as compared with free ART [39]. Results of the time spent in target quadrants, as presented in Figure 4C, confirm this finding and reveal the superiority of ART-loaded CS-coated SLNs over free ART. Time spent in the quadrant was 75.6 ± 0.87, 39.5 ± 0.64, 48.3 ± 0.54, and 61.2 ± 0.46 s for the negative, positive, ART, and CS-SLNs, respectively.

#### 2.9.3. Enzyme-Linked Immunosorbent Assay (ELISA)

**Figure 5:**
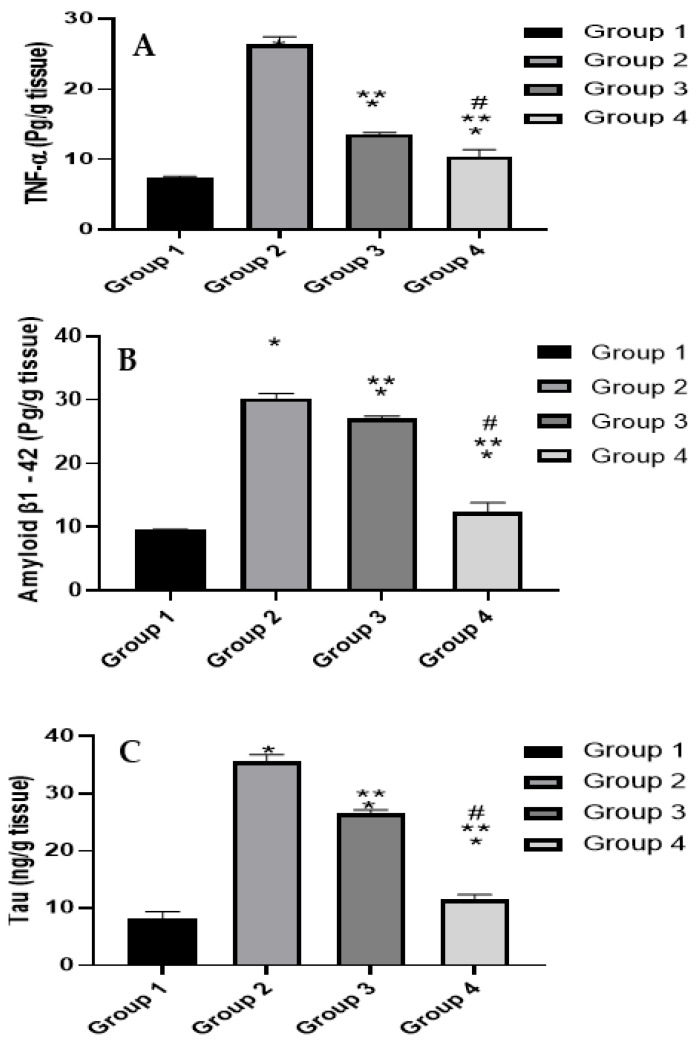
Biochemical analysis of (**A**) TNF-α level, (**B**) Amyloid β1-42 level and (**C**) Tau level. Statistical analyses are performed using one-way analysis of variance (ANOVA) followed by Tukey-Kramer post-hoc test, whereby each value was presented as mean ± standard deviation (SD). * Statistically significantly different from the normal control group (*p* < 0.05); ** statistically significantly different from the STZ group, 3 mg/kg (*p* < 0.05); # statistically significantly different from the ART group 50 mg/kg (*p* < 0.05).

The AD etiology is not yet fully clarified and many aspects contribute to its pathogenesis [52]. AD is characterized by the deposition of Aβ plaques [53,54]. Additionally, cytokines including TNF-α are actively participating in AD inflammatory response and were reported to accumulate in AD patients’ plasma and cerebrospinal fluid (CSF) [55]. Tau protein is among the most widely studied AD diagnostic markers where significant levels in CSF are linked to neuronal damage [56,57]. Levels of Aβ, TNF-α, and Tau protein were assessed after termination of the experiment and sacrifice of the animals and results are shown in Figure 5.

Induction of AD in the positive control (group 2) led to a substantial increase in levels of the inflammatory biomarker TNF-α, as well as Aβ and Tau levels compared with negative control (group 1). This is in accordance with reported data about this animal model [50]. ART treatment (group 3) was capable of significantly inhibiting the levels of TNF-α, an indication of the potential anti-inflammatory activity of the phytoconstituents in the brain tissue. However, a weaker (yet statistically significant) inhibitory effect was noticed for Aβ and Tau levels. This finding may prove beneficial where the phytoconstituents of the ART extract may have multiple targets that affect the pathological events that evolve in AD.

In agreement with the behavioral assessment results, ART-loaded CS-coated SLNs treatment (group 4) again showed superior therapeutic potential with the levels of TNF-α, Aβ, and Tau comparable to the negative control (group 1) and significantly lower in comparison to the positive control (group 2) and ART treated group (group 3). This finding again supports the multi-target therapeutic potential of ART on AD and the superior performance of the nano-encapsulated phytoconstituents in comparison to the free extract.

The treatment in this study with ART and its coated SLNs showed an interesting and promising break through the AD vicious cycle. AD is well known to be characterized by the pathologic aggregation of Aβ peptides and Tau hyperphosphorylation and accumulation, leading to chronic inflammation with elevated TNF-α level [58]. This elevation, in turn, is one of the main causes for exacerbation of Aβ and Tau levels, which further induce TNF-α level elevation, creating a vicious cycle responsible for progressive AD neurodegeneration [59,60]. The results showed a decrease in the Aβ and Tau levels in treatment groups, and consequently a possible decrease in neurological inflammation as evidenced by the decrease in TNF-α level. The profound decrease in TNF-α level is attributed to, at least in part, the significant decrease in Aβ and Tau levels. However, ART extract has antioxidant and anti-inflammatory activity, suggesting a possible direct inhibitory effect on TNF-α secretion. In this context, it may be possible to explain the remarkable decrease in TNF-α level in treatment groups in comparison to the less prominent (yet significant) decreases in Aβ and Tau levels.

#### 2.9.4. Histological Examination

**Figure 6:**
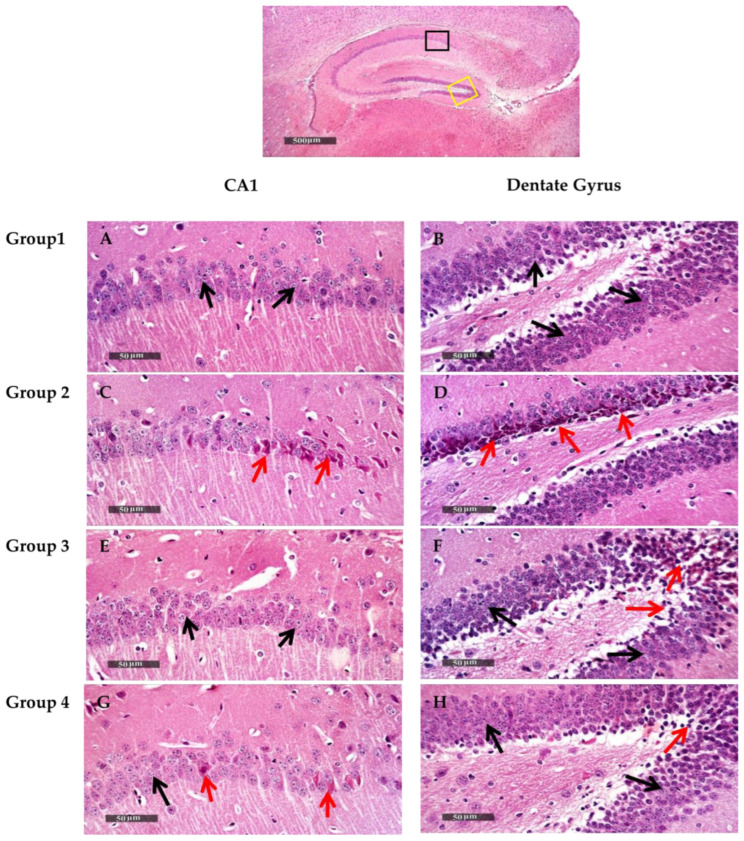
Anatomical overview of total hippocampal region highlighted examined fixed subregions in different groups (H&E stain, 40X; black boxed area for CA1 subregion and yellow boxed area for Dentate Gyrus subregion). Neuroprotective histological effect of different treatments on CA1 hippocampal subregions (left column micrographs) and Dentate Gyrus subregions (Right column micrographs) with correlated groups. H&E stain, 400X. Black arrows = intact neurons, red arrows = degenerated and necrotic neurons. (**A**,**B**) represent group1 (negative control). (**C**,**D**) represent group 2 (Positive control). (**E**,**F**) represent group 3 (ART). (**G**,**H**) represent group 4 (F3).

**Figure 7:**
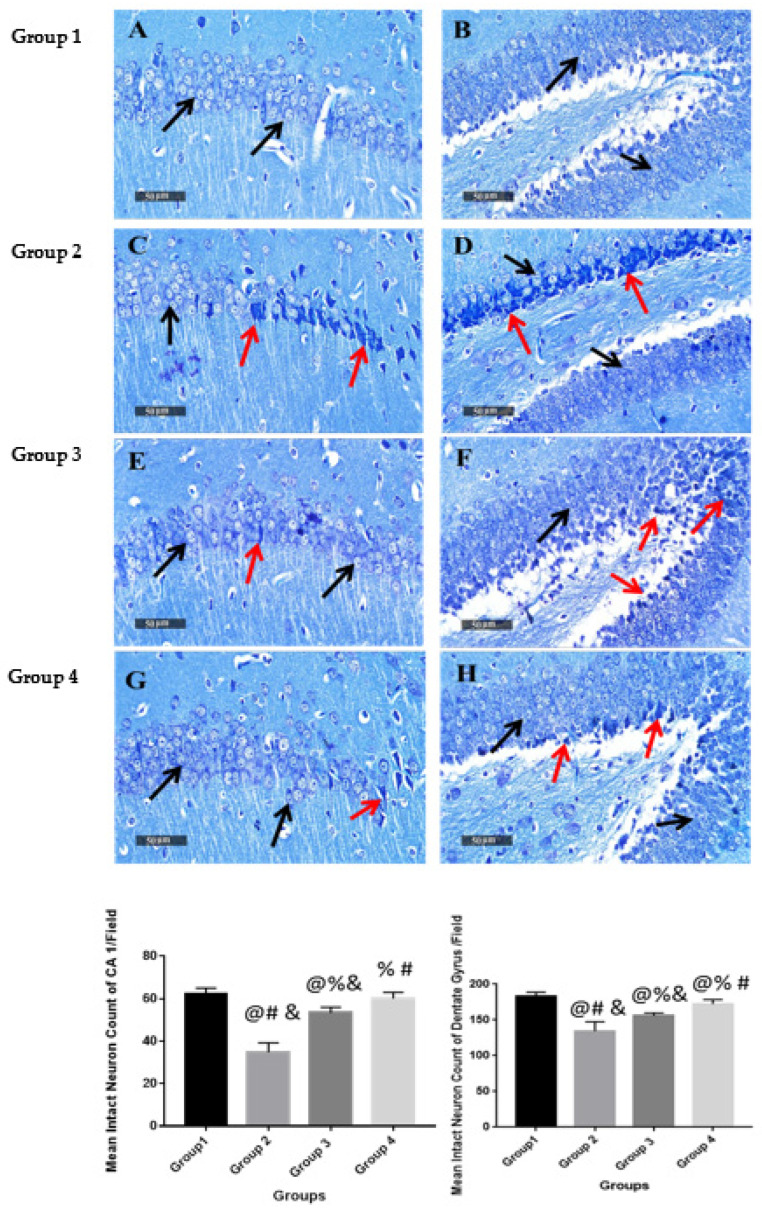
Light-microscopic examination of toluidine blue-stained pyramidal neurons in CA1 & Dentate Gyrus hippocampal sub regions in different groups with mean intact neuronal counts, 400X. Data were represented as mean ± SD (*n* = 6), black arrows = intact neurons, red arrows = damaged neurons. (**A**) and (**B**) represent group1 (negative control). (**C**) and (**D**) represent group 2 (Positive control). (**E**) and (**F**) represent group 3 (ART). (**G**) and (**H**) represent group 4 (F3). Statistical analyses were performed using one-way analysis of variance (ANOVA) followed by Tukey’s post hoc test. @ Significant as compared to negative control (group 1). % Significant as compared to positive control group (group 2). # Significant as compared to ART (group 3). & Significant as compared to ART formulation (group 4). Significant difference was conducted by one-way ANOVA at *p* < 0.0001.

Microscopic examination of hippocampal subregions CA1 and dentate gyrus was performed to assess the neurological changes accompanied by the different treatment modalities. The negative control (group 1) samples presented a normal organization and apparent intact morphological features of the hippocampal CA1 region. The CA1 subregion showed well organized pyramidal neurons with apparent intact subcellular and nuclear details (Figure 6A) with mean pyramidal intact neurons count in sections stained with toluidine blue of up to 62 cells/field (Figure 7A). Intact neuropil was noted with normally distributed glial cells together with normal vasculatures. However, in AD-induced mice (group 2), the CA1 subregion showed abundant neuronal damage, concurrent with many records of necrotic and hyperesenophilic shrunken neurons lacking their subcellular details (Figure 6C), and up to 46% neuronal loss in toluidine blue-stained sections (Figure 7C). Mild perineuronal edema and mild reactive glial cells infiltrates were recorded. Obvious neuroprotective efficacy with many apparent intact neurons was observed in the CA1 subregion of ART (group 3) treatment samples (Figure 6E) with occasional scattered neuronal degenerative changes and up to 35% more significant improvement of mean intact neurons count in Toluidine blue-stained tissue sections (Figure 7E) with respect to positive control (group 2). Moreover, ART-loaded CS-coated SLNs (group 4) treatment samples showed almost the same records as negative control samples with insignificant higher records of intact neurons count (Figure 6G) and single scattered records of neuronal damage (Figure 7G).

Microscopic examination of dentate gyrus subregions of negative control (group 1) samples showed almost well-organized, apparently intact granule cells neurons all over dentate gyrus blades (Figure 6B) with mean intact granule cells counting up to 182 cells/field in Toluidine blue-stained tissue sections (Figure 7B). Contrarily, in AD-induced (group 2) samples, dentate gyrus subregions displayed moderate records of neuronal degenerative changes of the inner small granule cell layer with nuclear pyknosis (Figure 6D), and significantly up to 27% neuronal loss compared with positive control samples (Figure 7D). Significant neuroprotective efficacy was shown in group 3 dentate gyrus subregions of different samples with focal records of damaged pyknotic neurons at dentate apex zone (Figure 6F), and up to 10% neuronal loss compared with negative control samples (Figure 7F). Moreover, group 4 treated samples demonstrated almost the same records and mean intact neurons count levels as group 3 samples (Figure 7H).

Our in vivo results suggest that ART rich in phenolic acids and flavonoid glycosides has the ability to exert neuroprotective effect against AD and to suppress its progression. This is in agreement with previously reported data regarding the ability of flavonoids to halt the progression of pathological symptoms of neurodegenerative diseases by interacting with several signaling protein pathways and modulating their actions. Flavonoids can stimulate vascular blood flow and inhibit neuronal apoptosis induced by neurotoxic elements, e.g., free radicals and β-amyloid proteins [61]. The ability of polyphenols to prevent in vitro amyloidogenesis and retard the progression of AD in vivo was previously reported [62,63]. The results of UPLC-ESI-MS/MS analysis showed that apigenin derivatives were the predominant components of artichoke extract. Interestingly, apigenin was reported to attenuate copper-mediated β-amyloid-induced neurotoxicity via its antioxidant and mitochondrial protective effects [64]. In addition, apigenin in an AD rat model was proven to inhibit glycogen synthase kinase-3 (GSK-3), which exerts a critical role in the hyper phosphorylation of tau protein and formation of Aβ plaques and neurofibrillary tangles [65]. Additionally, apigenin exhibited neuromodulator effect via microglial inactivation and over-expression of brain-derived neurotrophic factor (BDNF) in different AD models [66]. Moreover, caffeic acid was reported to exert dose-dependent antioxidant and neuroprotective properties via the modulation of critical signaling pathways [67,68]. Chlorogenic acid was shown to exhibit anti-acetylcholinesterase and anti-oxidant effects in different rat models of neural oxidative stress, e.g., scopolamine-induced amnesia [22], global cerebral ischemia-reperfusion [69], arsenite-induced neurotoxicity [70], and glutamate-induced excitotoxicity [71]. Further, rutin was reported to ameliorate the spatial memory, oxidative stress, and neuroinflammation in transgenic mice with AD through the reduction of Aβ-oligomer formation [72,73].

In addition, the in vivo study also clarifies the critical role of using a suitable delivery system for phytoconstituents to better exert their pharmacological activity, increase their aqueous solubility and absorption, and decrease their systemic loss through metabolism.

## 3. Material and Methods

### 3.1. Plant Material and Extraction

Fresh immature capitula of *Cynara cardunculus* L. were purchased from a local market early during the spring harvest (March 2021) and were helpfully authenticated by Mrs. Therese Labib, plant taxonomist at the Egyptian Ministry of Agriculture. A voucher specimen (PHG-P-CC-418) was conserved at the herbarium of Pharmacognosy Department, Faculty of Pharmacy, Ain Shams University (Cairo, Egypt). The outer and inner bracts were pulverized, macerated in methanol (1:6) for 48 h, and filtered. The extract was concentrated under vacuum (40 °C) using a rotary evaporator (Hei-VAP Value, Heidolph) and residual water was removed by lyophilization (Alpha 1-2 LD plus lyophilizer). The dried extract (ART) was kept at −20 °C until needed.

### 3.2. Assessment of Total Phenolic Content (TPC) and Total Flavonoids Content (TFC)

The Folin–Ciocalteu method was utilized to evaluate the TPC using gallic acid as standard according to previously described method [74]. The procedure starts with mixing 10 μL from sample/standard with 100 μL Folin-Ciocalteu reagent (diluted 1:10) in a 96-well microplate. Then, 80 μL of 1M Na_2_CO_3_ was added and kept for 20 min in the dark region at ambient temperature (25 °C). After incubation, a blue complex is produced and measured colorimetrically at 630 nm. Data are manipulated as means ±SD.

The aluminium chloride method was applied to assess the TFC, as previously reported [75] with minor modifications to be carried out in microplates. Briefly, 15 μL from sample/standard was added in microplate of 96-well type. Then, 175 μL methanol was added, followed by 30 μL of 1.25% AlCl3. Finally, 30 μL of 0.125 M C_2_H_3_NaO_2_ was added and incubated for 5 min. After incubation, a yellow complex is produced and measured colorimetrically at 420 nm. The data are manipulated as means ±SD.

### 3.3. UPLC-ESI-MS/MS Analysis

Chemical profiling of artichoke extract was performed by UPLC coupled to ESI-MS according to the recently published method [8]. Briefly, the sample was dissolved in HPLC grade methanol (100 μg/mL) and filtered through a membrane disc filter (0.2 μm). The injection volume was 10 μL. The UPLC (Acquity, Waters^®^, Milford, MA USA) was supplied with an Acquity UPLC-BEH C_18_ reversed-phase column (1.7 µm particle size, 2.1 × 50 mm). Mobile phase flow rate was set at 0.2 mL/min with a gradient elution program composed of acidified water (0.1% formic acid) and acidified methanol (0.1% formic acid) through a run of 35 min. The mass spectrometric analysis was accomplished on a XEVO TQD triple quadruple mass spectrometer (Waters Corporation, Milford, MA, USA) using ESI in negative ion acquisition mode under the following parameters: 30 eV cone voltage and 3 kV capillary voltage, at 150 °C source temperature and 440 °C desolvation temperature using vacuum pump by Edwards^®^ (Chandler, AZ, USA). Detection of mass spectra was in the range of 100–1000 *m*/*z* using Maslynx 4.1 and peaks were tentatively identified by comparing their mass spectra and fragmentation pattern with reported data.

### 3.4. Assessment of Antioxidant Properties

#### 3.4.1. DPPH Radical Scavenging Capacity

The 1,1-diphenyl-2-picryl-hydrazyl-hydrate (DPPH) free radical assay was performed according to a previously reported method [76]. Briefly, 100 μL of DPPH reagent (0.1% in methanol, freshly prepared) were added to the sample (100 μL) in 96-well plates (*n* = 6), and the reaction mixture was incubated at room temp for 20 min in dark. After incubation, the reduced intensity of DPPH colour was measured colorimetrically at 540 nm. The data are manipulated as means ± SD consistent with the following equation:

% Inhibition = [(Average absorbance of blank − average absorbance of the sample)/(Average absorbance of blank)] × 100

#### 3.4.2. Iron Metal Chelation Assay

The ferrozine iron metal chelation assay was carried out according to a previously described method [77] with minor modifications to be carried out in microplates. Briefly, 20 μL of the freshly prepared 0.3 mM ferrous sulphate were blended with 50 μL of the sample in a 96-well plate (*n* = 6). Afterwards, 30 μL of 0.8 mM ferrozine were added to each well. Afterwards, the reaction mixture was conserved for 10 min at ambient temperature. After incubation period, the reduced intensity of the produced complex was quantified colorimetrically at 562 nm. The data are manipulated as means ± SD following this equation:

% Inhibition = [(Average absorbance of blank − average absorbance of the sample)/(Average absorbance of blank)] × 100

#### 3.4.3. ABTS Decolorization Assay

The 2,2’-azino-bis(3-ethylbenzothiazoline-6-sulfonic acid (ABTS) radical cation decolorization assay was carried out according to a reported method [78], with minor modifications to be carried out in microplates. Briefly, 192 mg of ABTS were dissolved in distilled water and transferred into volumetric flask. Then, the volume was completed to 50 mL with distilled water. Hence, 1 mL of the previous solution was mixed with 17 μL from potassium persulphate (140 mM), and the mixture was maintained for one day in a dark region. After that, 1 mL of the reaction mixture was completed to 50 mL with methanol to reach the final dilution of ABTS dilution needed for this assay. Then, 190 μL of the previous prepared ABTS solution were mixed with the sample (10 μL) in 96-well plates (*n* = 4), the reaction was maintained for 2 hr in a dark area at ambient temperature. During the incubation period, the decreased intensity of ABTS colour was assessed colorimetrically at 734 nm. The data are manipulated as means ± SD as stated in the following equation:

% Inhibition = [(Average absorbance of blank − average absorbance of the sample)/(Average absorbance of blank)] × 100

#### 3.4.4. Oxygen Radical Absorbance Capacity (ORAC) Assay

The ORAC assay was established in accordance with the reported method of [79], with some modifications. In a brief, the prepared sample (10 mL) was kept along with 100 nM fluoresceine (30 μL) for 10 min at 37 °C. The fluorescence measurement (λ_ex_ 485 nm and λ_em_ 520 nm) was assessed for three runs (run time = 90 s) for background measurement. Afterward, 70 μL of freshly prepared 300 mM 2,2’-Azobis(2-amidinopropane) dihydrochloride (AAPH) were added immediately to each well. The fluorescence measurement (485 EX, 520 EM, nm) was continued for 60 min (40 runs, each run = 90 s). The data are manipulated as means ±SD in triplicate, the antioxidant effect of the extract was calculated as μM Trolox equivalents by substitution in the following linear regression equation:

Y = 377.7 × X + 29731 (R2 = 0.9972)

### 3.5. Preparation of Uncoated and Chitosan-Coated Artichoke-Loaded Solid Lipid Nanoparticles (SLNs)

Preparation of SLNs was carried out using a modified solvent emulsification evaporation method via w/o/w double emulsion technique by the method previously reported by Anchan et al. [39]. Briefly, the oil phase was prepared by dissolving 250 mg glycerol monostearate (GMS) in 2 mL chloroform in a stoppered glass tube in a bath sonicator. The sample was dissolved in 0.1 M HCl with mild shaking to form the internal aqueous phase (1 mg ART in 0.2 mL HCl), which was added to the oil phase and sonicated for 30 s in an ice bath to form w/o emulsion. Then, the formed emulsion was emptied in a beaker containing the external aqueous phase composed of either 2 % *w*/*v* Poloxamer 407 or Tween 80 and then sonicated for 30 s at 70% amplitude. The resultant w/o/w emulsion was stirred at 300 rpm using a magnetic stirrer to ensure complete organic solvent evaporation.

Preparation of chitosan-coated SLNs (CS-SLNs) was achieved by pouring the SLNs dispersion into a beaker containing chitosan (CS) (0.5% *w*/*v*) in 1% *w*/*v* acetic acid. The formed dispersion was then stirred overnight.

### 3.6. Measurement of Particle Size and Zeta Potential

The prepared formulations were analyzed for particle size, PDI, and ζ potential using Malvern zeta sizer (Nano ZS, Malvern Instruments, Malvern, UK). Formulations were first diluted by deionized water. Square glass cuvettes were used for particle size measurements, while clear disposable zeta cells were used for ζ potential. All measurements were performed in triplicate.

### 3.7. Measurement of Entrapment Efficiency (EE%)

Centrifugal ultrafiltration via Centrisart^®^-I tube (MWCO 300 kDa, Sartorius AG, Goettingen, Germany) was performed to separate SLNs from supernatant containing excess unentrapped ART. Unentrapped ART concentration in the filtrate was determined spectrophotometrically at 330 nm and % EE was calculated using the following equation

$$\%\;EE\;\left(indirect\right)=\frac{Total\;ART\;concentration-concentration\;of\;unencapsulated\;ART\;}{Total\;ART\;concentration}\;\times \;100$$

The experiment was run in triplicates and results were expressed as means ± SD.

### 3.8. Morphological Examination by Transmission Electron Microscopy (TEM)

The morphology of selected uncoated and coated SLNs prepared with Poloxamer 407 was examined by TEM (JEM-100CX, JEOL, Tokyo, Japan) after staining by uranyl acetate.

### 3.9. In Vitro Release Study

An *in vitro* release test was carried out in 0.1 N HCl solutions using the dialysis method. Briefly, uncoated CS-SLNs and ART were put in dialysis bags (Spectra/Por^®^ membrane MWCO 100,000 Spectrum) which were subsequently sealed with medicell clips (Spectrum) at both ends, fixed to paddle shafts in USP dissolution apparatus (Pharma Test, Hainburg, Germany). They were then suspended in 0.1 N HCl pH 1.2 to ensure sink conditions. The experiment was performed at 37 ± 0.5 °C and at 100 rpm agitation speed. Samples were taken at fixed time intervals and replaced with fresh media. ART concentrations were determined spectrophotometrically at 330 nm. The experiment was carried out in triplicate and results are expressed as means ±SD. To analyze the release kinetics of the studied samples, release data were fitted into different models, namely zero-order, first order, and Higuchi model using DD solver software.

### 3.10. Ex Vivo Permeation Study Using Goat Intestinal Mucosa

For this experiment, fresh goat intestinal mucosa was supplied by the local slaughterhouse, trimmed off for any extraneous tissues then washed with phosphate buffered saline (pH 6.8) to eliminate lumen contents and mucous. Then, it was placed in a Franz diffusion cell between donor and receptor compartments so that the mucosal surface faces the donor compartment. ART, uncoated, and CS-SLNs (equivalent to 50 µg/mL ART) were positioned in the donor compartment, whereas 25 mL phosphate buffer (pH 6.8) was added to the receptor part and kept at 100 rpm and 37 °C. Aliquots of 2 mL were taken at fixed time intervals from the receptor compartment and compensated with fresh buffer and concentration of ART was assessed spectrophotometrically at 330 nm. The experiment was run in triplicates and results were expressed as mean ± SD.

### 3.11. In Vivo Study

#### 3.11.1. Animals

Animal experiments were accomplished under approval and compliance of the ethical guidelines of Ethics Committee for the use of animal subjects, Faculty of Pharmacy, Ain Shams University, Cairo, Egypt (approval number 105) which comply with the ARRIVE guidelines and the EU Directive 2010/63/EU for animal experiments. Adult male Swiss Albino mice weighing 20 ± 2 g were obtained from the animal house, National Research Center (Cairo, Egypt), and were left for 1 week for adaptation (4–5/cage), under controlled temperature and humidity with light/dark cycles of 12 h and ad libitum access to food and water.

#### 3.11.2. Induction of AD and Treatment Modalities

The induction of AD was carried out by the freehand intracerebroventricular (ICV) procedure using streptozotocin (STZ) as formerly described by Sorial et al. [50]. At day zero, the animals were divided into 4 groups (*n* = 8). Group 1 was the normal control group which received saline whereas AD was induced in groups 2, 3 and 4 which received STZ at 3 mg/kg [80] thorough ICV injection. Briefly, mice were anesthetized with ether and the head was stabilized using downward pressure above the ears, and the needle was inserted directly through the skin and skull into the lateral ventricle. The lateral ventricle was targeted by visualizing an equilateral triangle between the eyes and the center of the skull to locate the bregma, allowing the needle to be inserted approximately 1 mm lateral to this point. Mice displayed normal behavior within 1 min following the injection. Groups 3 and 4 were followed by the oral administration of treatment (ART and selected ART formulation F3; equivalent to ART 50 mg/kg, daily for 21 days), respectively, in comparison to saline for group 2. At day 22, mice were euthanized via cervical dislocation. Then, the hippocampus was separated to perform the biochemical analyses, while the whole brain was kept for histopathological examination.

#### 3.11.3. Behavioral Assessment of the Effects of ART

##### Y-Maze

The Y-maze test was accomplished as described by Sorial et al. [50] and Abbas et al. [52] to assess the animal short-term memory on the last 2 days of treatment. The apparatus comprised a 3-arm metallic Y-maze. The experiment was accomplished in 8-min sessions for 2 consecutive days. Olfactory cues that can entail errors in observations were eliminated by cleaning the maze with 70% ethanol after each mouse. The actual alternation is identified as consecutive entries into all 3 arms, referred to as overlapping triplet sets, whereas possible alternation is identified as the total number of arm entries. Spontaneous alternation behavior percentage is determined as the ratio of actual alternations/possible alternations × 100. The results were expressed as means ± SD.

##### Morris Water Maze (MWM)

The experiment is reported [50,52] to evaluate animals visuospatial memory and learning capacity. Mice were subjected to training trials for 4 successive days (2 trials/day, maximum trial time = 120 s) at the last 4 days of treatment. The escape latency is determined as the average total time taken to find the platform during the two training sessions on every acquisition day. This is utilized as an acquisition learning index. During the 5th day, mice underwent a probe-trial session where the platform was removed from the pool. Every mouse was permitted to explore the pool for a period of 60 s. The time taken by every mouse in the target quadrant, where the platform was formerly placed, is used as an index of retrieval or memory. Results were expressed as mean ± SD.

#### 3.11.4. Enzyme-Linked Immunosorbent Assay (ELISA)

Brains of the animals (*n* = 8) in each group were recovered and homogenized (10% homogenate in 0.1 M PBS), then centrifuged. The supernatants were kept for further analyses. Amyloid beta peptide (Aβ1-42) MyBioSource, San Diego, CA, USA), TNF-α (MyBioSource, San Diego, CA, USA) as well as Tau protein (NOVUS biological, Littleton, CO, USA) content in the brains were quantified utilizing commercially available ELISA kits following manufacturers’ instructions. The results were expressed as means ± SD.

#### 3.11.5. Histopathology

Dissected rat brain tissues were washed then fixed in 10% neutral buffered formalin for 72 h. Samples were then processed in different grades of ethanol, subsequently cleared in Xylene, and infiltrated with Paraplast Plus tissue embedding media (Leica biosystems). Hence, 4-μm thick serial sagittal brain sections were prepared by rotatory microtome for the examination of hippocampal subregions and finally mounted on glass slides where they were stained by Hematoxylin and Eosin for pathological examination. Another set of tissue sections was stained by Toluidine blue stain for the quantification of intact neurons, then inspected with light microscope. Standard protocols for sample preparation, staining, and analysis were followed according to Abbas, Culling and Abbas [81,82,83].

## 4. Conclusions

Artichoke bracts represent a promising, multi-targeted, botanical approach in AD therapy. The phytochemical profiling of artichoke bracts extract revealed richness in caffeoylquinic acids and flavonoid glycosides of apigenin, luteolin, kaempferol, and quercetin, which confirms the remarkable total phenolic and flavonoid contents. Broad antioxidant spectrum was established in vitro via different mechanisms. The anti-Alzheimer potential of artichoke bracts extract and its chitosan-coated solid lipid nanoparticles was studied in vivo in a streptozotocin-induced Alzheimer mice model. A marked enhancement of memory and cognitive functions and a significant reduction of the levels of inflammatory markers and the AD pathogenesis hallmarks, β-amyloid and tau proteins, were observed together with histopathological signs of neuroprotective efficacy in dentate Gyrus sub-regions of mice treated with artichoke extract and to a greater extent with chitosan-coated artichoke-loaded solid lipid nanoparticles. Further studies are in progress with the final aim to propose a botanical treatment for Alzheimer’s disease. Our findings will alter the future medicinal outlook of artichoke bracts, previously regarded as agro-industrial waste.

## Figures and Tables

**Table 1:** Metabolite profiling of artichoke bracts methanolic extract (ART) as analyzed by UPLC-ESI-MS/MS.

No	t_R_ (min)	[M − H]^−^ *m*/*z*	Molecular Weight	MS^(*n*)^ *m*/*z*	Molecular Formula	Tentative Identification	Class	Reference(s)
**1.**	0.75	178	179	135	C_9_H_8_O_4_	Caffeic acid	Phenolic acid	[23]
**2.**	0.98	181	182	155, 140	C_9_H_10_O_4_	Syringaldehyde	Aldehyde	[24]
**3.**	1.15	353	354	191, 179, 135	C_16_H_18_O_9_	Chlorogenic acid	Phenolic acid	[24]
**4.**	13.72	515	516	353, 179, 191	C_25_H_24_O_12_	1,3 di-*O*-caffeoylquinic acid	Phenolic acid	[24]
**5.**	13.90	515	516	335, 317, 203, 179, 191	C_25_H_24_O_12_	3,4 di-*O*-caffeoylquinic acid	Phenolic acid	[25]
**6.**	18.31	521	522	359, 223, 161	C_24_H_26_O_13_	Rosmarinic acid-*O*-hexoside	Phenolic acid glycoside	[26]
**7.**	19.09	415	416	269	C_21_H_20_O_9_	Apigenin-*O*-deoxyhexoside	Flavonoid glycoside	[27]
**8.**	19.47	577	578	269, 225, 151	C_27_H_30_O_14_	Apigenin-*O*-rutinoside	Flavonoid glycoside	[23]
**9.**	20.02	445	446	269, 151, 117	C_21_H_18_O_11_	Apigenin-*O*-hexouronide	Flavonoid glycoside	[25]
**10.**	20.98	447	448	285, 241, 151	C_21_H_20_O_11_	Luteolin-*O*-hexoside	Flavonoid glycoside	[28,29]
**11.**	22.12	593	594	285, 227, 151	C_27_H_30_O_15_	Kaempferol-*O*-rutinoside	Flavonoid glycoside	[28,29]
**12.**	22.35	283	284	270, 229, 225	C_16_H_12_O_5_	Glycitein	Flavonoid glycoside	[28,29]
**13.**	23.39	609	610	301, 300, 271, 243, 227	C_27_H_30_O_16_	Quercetin-3-*O*-hexoside-3-deoxyhexoside (Rutin)	Flavonoid glycoside	[12]
**14.**	24.54	431	432	269, 225, 151, 117	C_21_H_20_O_10_	Apigenin-*O*-hexoside	Flavonoid glycoside	[27]
**15.**	31.02	533	534	490, 489, 285	C_25_H_25_O_12_	Luteolin-*O*-diacetylhexoside	Flavonoid glycoside	[26]
**16.**	31.72	683	684	341, 179, 143, 131	C_15_H_18_O_9_	Caffeic acid-*O*-hexoside dimer	Phenolic acid glycoside	[26]

**Table 2:** SLNs composition, particle size, PDI, EE % and release order.

Formulation Code	F1	F2	F3	F4
**Internal aqueous phase**	ART (1 mg) in 0.2 mL 0.1 M HCl			
**Lipid phase**	GMS			
**External aqueous phase**	Poloxamer 407	Tween 80	Poloxamer 407	Tween 80
**CS coating solution (%*w*/*v*)**	-------		0.50%	
**Particle size (nm)**	165.3 ± 1.69	320.4 ± 1.03	198.3 ± 1.98	448.3 ± 1.23
**PDI**	0.235 ± 0.006	0.312 ± 0.004	0.203 ± 0.003	0.504 ± 0.0031
**Zeta potential (mV)**	−26.3 ± 2.04	−32.5 ±1.45	19.25 ±1.89	22.4 ±0.87
**EE %**	74 ± 1.56	57 ± 2.06	79.2 ± 1.12	54 ± 1.09
**Release fitting (R2)**				
**Zero order**	0.947	0.941	0.945	0.959
**First order**	0.982	0.987	0.908	0.9344
**Higuchi**	0.988	0.984	0.964	0.975

## Data Availability

Data is contained within the article and supplementary material.

## Supplementary Materials

The following supporting information can be downloaded at: https://www.mdpi.com/article/10.3390/ph15101202/s1, Figure S1: Total ion chromatogram (TIC) of the methanolic extract of *C. cardunculus* L. bracts.
