# Supplementary material for: Neuroprotective Effect of Artichoke-Based Nanoformulation in Sporadic Alzheimer’s Disease Mouse Model: Focus on Antioxidant, Anti-Inflammatory, and Amyloidogenic Pathways
Source: Pharmaceuticals (Basel). 2022 Sep 28;15(10):1202. doi: 10.3390/ph15101202 (PMC9610800; doi:10.3390/ph15101202)
Supplement: Supplementary file 1 [file pharmaceuticals-15-01202-s001.zip › pharmaceuticals-1879646-supplementary.pdf]

Neuroprotective Effect of Artichoke-Based Nanoformulation in Sporadic Alzheimer's Disease Mouse Model: Focus on Antioxidant, Anti-Inflammatory, and Amyloidogenic Pathways

Heba A. S. El-Nashar [1], Haidy Abbas [2], Mariam Zewail [2], Mohamed H. Noureldin [3], Mai M. Ali [4], Marium M. Shamaa [3], Mohamed A. Khattab [5] and Nehal Ibrahim [1,*]

[1] Pharmacognosy Department, Faculty of Pharmacy, Ain Shams University, Cairo 11566, Egypt; heba_pharma@pharma.asu.edu.eg (H.A.S.E.-N.); nehal.sabry@pharma.asu.edu.eg (N.I.)
[2] Department of Pharmaceutics, Faculty of Pharmacy, Damanhour University, Damanhour *22514,* Egypt; haidy.abass@pharm.dmu.edu.eg (H.A.); mariamzewail@pharm.dmu.edu.eg (M.Z.)
[3] Department of Biochemistry, Division of Clinical and Biological Sciences, College of Pharmacy, Arab
Academy for Science, Technology and Maritime Transport, Alexandria P.O. Box 1029, Egypt; mohamed.noureldin@aast.edu (M.H.N.); marium.muhamed@aast.edu (M.M.S.)
[4] Department of Pharmaceutics, Division of Pharmaceutical Sciences, College of Pharmacy, Arab Academy for Science, Technology and Maritime Transport, Alexandria P.O. Box 1029, Egypt; drmaiali@aast.edu (M.M.A.)
[5] Department of Cytology and Histology, Faculty of Veterinary Medicine, Cairo University, Cairo 12211, Egypt; mabdelrazik@cu.edu.eg
* Correspondence: nehal.sabry@pharma.asu.edu.eg

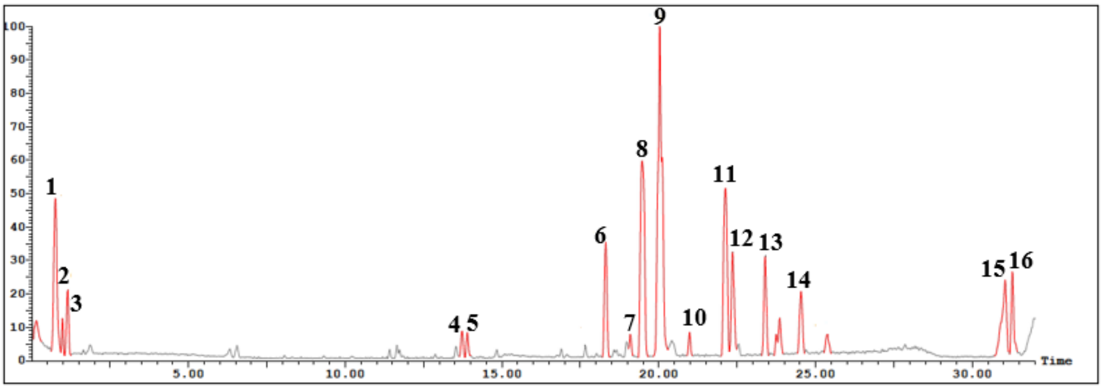

Figure S1. Total ion chromatogram (TIC) of the methanolic extract of *C. cardunculus* L. bracts. Peaks numbers follow those listed in Table 1.
